# Genome-Wide Association Study of Microscopic Colitis in the UK Biobank Confirms Immune-Related Pathogenesis

**DOI:** 10.1093/ecco-jcc/jjz104

**Published:** 2019-05-24

**Authors:** Harry D Green, Robin N Beaumont, Amanda Thomas, Benjamin Hamilton, Andrew R Wood, Seth Sharp, Samuel E Jones, Jessica Tyrrell, Gareth Walker, James Goodhand, Nicholas A Kennedy, Tariq Ahmad, Michael N Weedon

**Affiliations:** 1 Genetics of Complex Traits, University of Exeter Medical School, Exeter, UK; 2 IBD Pharmacogenetics, Royal Devon and Exeter NHS Foundation Trust, Exeter, UK

**Keywords:** Microscopic colitis, genetics, inflammatory bowel disease, ulcerative colitis, Crohn’s disease, genome wide association study

## Abstract

**Background and Aims:**

The causes of microscopic colitis are currently poorly understood. Previous reports have found clinical associations with coeliac disease and genetic associations at the human leukocyte antigen [HLA] locus on the ancestral 8.1 haplotype. We investigated pharmacological and genetic factors associated with microscopic colitis in the UK Biobank.

**Methods:**

In total, 483 European UK Biobank participants were identified by ICD10 coding, and a genome-wide association study was performed using BOLT-LMM, with a sensitivity analysis performed excluding potential confounders. The HLA*IMP:02 algorithm was used to estimate allele frequency at 11 classical HLA genes, and downstream analysis was performed using FUMA. Genetic overlap with inflammatory bowel disease [Crohn’s disease and ulcerative colitis] was investigated using genetic risk scores.

**Results:**

We found significant phenotypic associations with smoking status, coeliac disease and the use of proton-pump inhibitors but not with other commonly reported pharmacological risk factors. Using the largest sample size to date, we confirmed a recently reported association with the MHC Ancestral 8.1 Haplotype. Downstream analysis suggests association with digestive tract morphogenesis. By calculating genetic risk scores, we also report suggestive evidence of shared genetic risk with Crohn’s disease, but not with ulcerative colitis.

**Conclusions:**

This report confirms the role of genetic determinants in the HLA in the pathogenesis of microscopic colitis. The genetic overlap with Crohn’s disease suggests a common underlying mechanism of disease.

## 1. Introduction

Microscopic colitis includes two related inflammatory bowel disorders, lymphocytic colitis and collagenous colitis that have a combined prevalence of 103 cases per 100 000 population.^1^ Both disorders cause chronic watery non-bloody diarrhoea and incontinence, and are associated with normal endoscopic appearances and characteristic histological features. The primary histological feature of lymphocytic colitis is patchy lymphocytic infiltration of the epithelium with preserved crypt architecture. Collagenous colitis is characterized by the presence of a thickened subepithelial collagen layer.

The pathogenesis of microscopic colitis is poorly elucidated: it reportedly involves immune responses to luminal factors in genetically predisposed individuals.^2^ A recent association study based on Immunochip data reported association between human leukocyte antigen [HLA] alleles on the 8.1 haplotype and collagenous colitis^3^ but not with lymphocytic colitis^4^ in cohorts that comprised 314 patients with collagenous colitis, 122 patients with lymphocytic colitis and 4299 controls. Furthermore, Westerlind *et al*. report genetic overlap with inflammatory bowel disease [IBD] by comparing the number of nominally significant single nucleotide polymorphisms [SNPs] in both phenotypes.^3^ The most frequently cited environmental risk factors are medications, with non-steroidal anti-inflammatory drugs [NSAIDs], proton-pump inhibitors [PPIs] and selective serotonin reuptake inhibitors [SSRIs] most commonly implicated.^5–7^ There have been no reported investigations of potential pharmacogenetic risk factors for microscopic colitis.

We sought to identify phenotypic and genetic associations with microscopic colitis in individuals of European ancestry enrolled in the UK Biobank, and report subsequent downstream analysis. Subsequently, we stratified the data by drug use and performed a genome-wide association study [GWAS] to identify pharmacogenetic associations. Finally, we calculated genetic risk scores for IBD to quantify in detail genetic overlap with microscopic colitis.

## 2. Methods

### 2.1. Participants

The UK Biobank is a population-based prospective study comprising more than 500 000 UK participants aged 40–69 years at time of recruitment between 2006 and 2010. Participants are actively followed, and phenotypic data collected include demographics, medical conditions, medications, lifestyle and anthropometric measurements. SNP genotypes were generated from the Affymetrix Axiom UK Biobank array [∼450 000 individuals] and the UK BiLEVE array [∼50 000 individuals]. More detail on the UK Biobank can be found elsewhere.^8^

We defined microscopic colitis by the ICD10 code K52.8. The full UK Biobank dataset contains 522 K52.8 cases and 502 097 controls [104.0/100 000 persons]. We stratified the data to contain only white Europeans by performing principal component analysis in the 1000 Genomes reference panel. Of the 451 099 participants remaining, 483 are cases and 450 616 are controls [107.2/100 000 persons]. Of the 483 cases, 335 [69.4%] had K52.8 as their primary diagnosis code.

We investigated pharmacological associations with NSAIDs, PPIs and SSRIs using the medication codes reported in the UK Biobank [Data-Field 20003: Treatment/medication code]. Drugs included and their relative frequencies in the UK Biobank among cases and controls are detailed in Supplementary Table 1.

### 2.2. Statistical Methods

We performed tests for association with clinical characteristics using the Mann–Whitney U test for continuous data and Fisher’s exact test for categorical data. Probability (*p*) values are reported uncorrected for multiple testing, but we used an adjusted *p*-value threshold of 0.0045 for statistical significance for the clinical data.

Quality control of the genotype data was performed centrally by the UK Biobank.^9^ For the GWAS, we used ~12.0 million Haplotype Reference Consortium [HRC] imputed variants with an imputation *r*^2^ ≥ 0.9, minor allele frequency [MAF] ≥ 0.025 [2.5%] and with a Hardy–Weinberg equilibrium *p* > 1 × 10^–12^. Furthermore, individuals with IBD or coeliac disease were excluded from all genetic analyses, leaving 423 cases and 445 232 controls.

We performed our main association test using BOLT-LMM v2.3,^10^ which applies a linear mixed model [LMM] to adjust for the effects of population structure and individual relatedness and allowed us to include all related individuals in our white European subset, rather than reducing the sample size to only include the unrelated individuals [379 768]. Covariates included were age, sex, recruiting centre and genotyping chip. A more detailed explanation of the GWAS methodology can be found in our recent publication,^11^ and a principal components plot is available in Supplementary Figure 1. Odds ratios from BOLT-LMM were calculated by $OR=e^{\beta \;/\;(\mu \;*\;(1\;-\;\mu ))}$ where μ = case fraction, and standard errors were divided by (μ ∗ (1 − μ)) to give confidence intervals. Following the GWAS, FUMA’s SNP2GENE analysis was used to convert SNP data into genomic loci, and to perform a gene-set enrichment analysis using MAGMA.^12^

As a sensitivity analysis we also performed a secondary GWAS on a more refined phenotype using only white British unrelated individuals, in which we excluded coeliac and IBD participants [defined by ICD10, ICD9 and self-report] and participants using PPIs. Controls were further refined by excluding those with a diagnosis [self-reported or in Hospital Episode Statistics data] before the age of 70 years of coronary heart disease, stroke, diabetes, chronic obstructive pulmonary disease, renal failure, any cancer [excluding non-melanoma skin cancer], and those who had died from any cause before the age of 70 years. To further limit the possibility of type 1 errors, this GWAS was performed using Fisher’s exact test. This analysis used 335 cases and 64 300 controls [253 cases and 49 608 controls following filtering of related individuals].

Imputation of HLA alleles was performed using the HLA*IMP:02 algorithm to estimate allele frequency at 11 classical HLA genes: HLA-A, -B, -C, -DRB5, -DRB4, -DRB3, -DRB1, -DQB1, -DQA1, -DPB1 and -DPA1 using reference panels described by Motyer *et al*.^13^ This procedure provides accurate dosages for 362 SNPs in the HLA region, allowing identification of the genetic basis of autoimmune processes with greater precision.^14^

Genetic risk scores for ulcerative colitis [UC], Crohn’s disease [CD] and IBD were calculated using odds ratios [ORs] for previously published SNPs.^15^ From that study, using only SNPs that were genome-wide significant at 5 × 10^–8^ and also present in our imputation panel, we have 145 SNPs for CD, 89 for UC and 162 for IBD. To quantify genetic overlap, for each IBD phenotype, we compared the mean genetic risk score (GRS) among the microscopic colitis patients with controls [defined as not having UC, CD, IBD or microscopic colitis]. A GRS containing N SNPs was calculated according to the equation below where β is the β-coefficient [log OR] representing the association between each SNP and the relevant phenotype and $d_{i}$ is the estimated dosage. Statistical associations are quantified using a two-tailed *t*-test.

$$\begin{array}{l} GRS=\underset{i=1}{\overset{N}{\sum }}\;\beta_{i}\cdot d_{i} \end{array}$$

## 3. Results

### 3.1. Clinical Associations

Demographic and drug associations are shown in Table 1. Microscopic colitis patients were more likely to be older, female and a current smoker. White European UK Biobank participants with microscopic colitis had an eight times higher risk of coeliac disease and 12 times higher risk of IBD than controls. We found no evidence of association with body mass index [BMI] or socioeconomic status [defined by the Townsend Deprivation Index].

**Table 1.** Clinical associations with microscopic colitis

Variable	Cases	Controls	*p*-value
**Age at recruitment [years]**	**61.9 [56.2–65.4]**	**58.6 [50.5–63.8]**	**5 × 10** ^**–15**^
Body mass index	26.6 [23.7–29.6]	26.7 [24.1–29.9]	0.195
Townsend Deprivation Index	−2.03 [−3.55 to 0.28]	−2.27 [−3.70 to 0.23]	0.171
**Female**	**65.6% [317/483]**	**54.2% [244 531/450 616]**	**5 × 10** ^**–7**^
**Current smoker**	**14.7% [71/483]**	**10.4% [46 792/450 616]**	**0.003**
**Coeliac disease**	**3.3% [16/483]**	**0.4% [1991/450 616]**	**7 × 10** ^**−10**^
**IBD**	**9.5% [46/483]**	**0.8% [3896/450 616]**	**2 × 10** ^**−32**^
**Using PPIs**	**20.3% [98/483]**	**10.3% [46 397/450 616]**	**9 × 10** ^**−11**^
Using SSRIs	5.8% [28/483]	4.0% [18 003/450 616]	0.048
Using NSAIDs	28.4% [137/483]	26.3% [118 687/450 616]	0.326
Using statins	19.7% [388/483]	16.3% [73 365/450 616]	0.048

The table shows demographic and drug use associations in the UK Biobank. Continuous variables are reported in terms of median [interquartile range]. All *p* values were computed by a Fisher’s or Mann–Whitney U test. A *p* value threshold was 0.0045 after Bonferroni correction. Significant associations are in bold type. Abbreviations: IBD, inflammatory bowel disease; NSAIDs, non-steroidal anti-inflammatory drugs; SSRIs, selective serotonin reuptake inhibitors; PPIs, proton-pump inhibitors.

In terms of drug associations, patients with microscopic colitis were twice as likely to be using PPIs [a sub-analysis suggests 1.95 for omeprazole, 2.00 for lansoprazole]. We found no significant associations for SSRIs, NSAIDs or statins when accounting for multiple testing. We also performed a sub-analysis of NSAIDs, stratifying by COX1 and COX2 inhibitors, but found no significant associations. All clinical associations were robust to adjustment for age and sex in a multivariable logistic regression model, and testing with or without the related individuals.

### 3.2. GWAS Results

**Figure 1.**
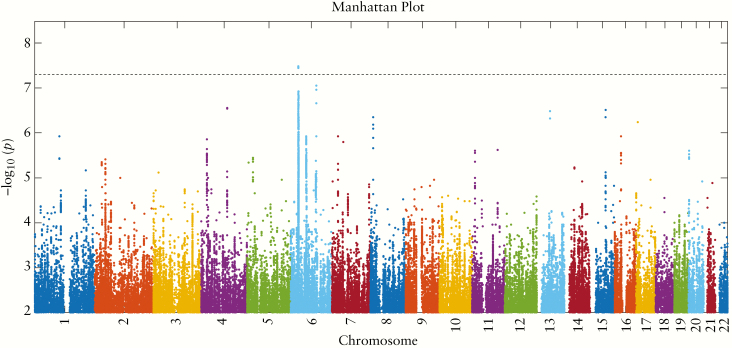
Manhattan plot showing the –log_10_[*p*] values for each single nucleotide polymorphism [SNP] in the HRC Imputation Panel with *p* < 0.01, computed using Bolt-LMM and plotted using an in-house MATLAB script which we have made publicly available on the MATLAB File Exchange.^16^ The horizontal dashed line is the genome-wide significance threshold at *p* = 5 × 10^–8^. The strongly significant signal on chromosome 6 lies in the HLA region, with lead SNP rs2596560, *p* = 3 × 10^–8^.

Figure 1 shows a Manhattan plot of all SNPs that passed quality control and had *p* < 0.01. We found a strong association for microscopic colitis on chromosome 6, in the HLA region, with lead SNP rs2596560 [OR 0.64, 95% confidence interval 0.56–0.72, *p* = 3×10^–8^, MAF 0.24 vs 0.33]. The odds ratio remained the same at the lead SNP [OR 0.64, 95% confidence interval 0.53–0.77, *p* = 3.3 × 10^–6^] when we performed our secondary analysis, on the refined phenotype using Fisher’s exact test.

Summary statistics for all genomic risk loci with *p* < 10^–5^ are available in Supplementary Table 2, and the full GWAS summary statistics can be found at https://www.ebi.ac.uk/gwas/home. In Supplementary Figure 2 we present a QQ plot. The genomics inflation factor λ was 1.0000. We also include the top ten results of a MAGMA Gene-set Analysis in Supplementary Table 3, in which we find a Bonferroni-significant association for digestive tract morphogenesis. ^17^

HLA imputation demonstrated that the SNP rs2596560 associates with the class I and II alleles that comprise the ancestral major histocompatibility [MHC] 8.1 haplotype previously been linked to microscopic colitis, with lead SNP B_0801 passing genome-wide significance. Results from BOLT-LMM runs on the HLA imputed alleles are given in Table 2, with alleles on the MHC 8.1 haplotype highlighted in bold.

**Table 2.** Classical HLA alleles

SNP	Allele frequency	Beta	SE	*p*-value
**B_801**	**0.143**	**4.3 × 10** ^**–4**^	**9.4 × 10** ^**–5**^	**3.9 × 10** ^**–6**^
**DRB1_301**	**0.147**	**3.7 × 10** ^**–4**^	**9.2 × 10** ^**–5**^	**5.6 × 10** ^**–5**^
DRB3_9901	0.655	−2.6 × 10^–4^	6.9 × 10^–5^	1.5 × 10^–4^
**DRB3_101**	**0.166**	**3.3 × 10** ^**–4**^	**8.8 × 10** ^**–5**^	**2.0 × 10** ^**–4**^
**DQA1_501**	**0.230**	**2.9 × 10** ^**–4**^	**7.8 × 10** ^**–5**^	**2.1 × 10** ^**–4**^
**C_701**	**0.175**	**3.2 × 10** ^**–4**^	**8.6 × 10** ^**–5**^	**2.4 × 10** ^**–4**^
**DQB1_201**	**0.148**	**3.4 × 10** ^**–4**^	**9.2 × 10** ^**–5**^	**2.7 × 10** ^**–4**^

The table shows the allele frequencies, beta, standard error and *p* values of all classical HLA alleles with a *p* value below 5 × 10^–4^ [computed through Bolt-LMM] and MAF > 0.01. SNPs in bold are part of the HLA A1-B8-DR3-DQ2 [ancestral MHC 8.1] haplotype.

To follow up on the phenotypic association with PPIs, we sought to identify pharmacogenetic associations by using PPIs as an inclusion criterion [comparing PPIs with microscopic colitis and PPIs with no microscopic colitis]. However, there were no significant genome-wide associations for this GWAS.

### 3.3. Genetic Overlap with IBD

Table 3 shows the mean CD, UC and IBD genetic risk scores and 95% confidence intervals for microscopic colitis patients and controls.

**Table 3.** Genetic risk score means and associations: microscopic colitis patients are significantly enriched for genetic risk factors for Crohn’s disease and IBD, but not for ulcerative colitis

Disease for which genetic risk score derived	Mean [95% CI] score for microscopic colitis patients	Mean [95% CI] score for controls	*p* value
Crohn’s disease	0.9688 [0.9632–0.9739]	0.9634 [0.9632–0.9636]	0.035
Ulcerative colitis	0.9928 [0.9859–0.9997]	0.9890 [0.9888–0.9892]	0.261
IBD	0.9286 [0.9237–0.9335]	0.9230 [0.9229–0.9231]	0.019

Abbreviation: IBD, inflammatory bowel disease.

Microscopic colitis patients had a higher genetic risk for all three tests, but only CD [*p* = 0.035] and IBD [*p* = 0.019] were significant at the 5% confidence level, suggesting some shared genetic pathway behind microscopic colitis and CD/IBD. These were robust to applying the same tests on only the unrelated individuals in the UK Biobank as a sensitivity analysis. In Table 4 we show which of the known risk loci replicate at *p* < 0.05 for microscopic colitis, although none of these are significant when Bonferroni correcting the *p* value threshold.

**Table 4.** Nominally significant SNPs in Crohn’s disease and IBD GRS

GRS	Chromosome	Position	A1	A1 frequency	Beta	SE	*p*
Both	10	6081230	C	0.836092	2.2 × 10^–4^	8.85 × 10^–5^	1.4 × 10^–2^
Both	2	28614794	C	0.519615	1.5 × 10^–4^	6.58 × 10^–5^	1.9 × 10^–2^
Both	22	30493882	G	0.55509	1.8 × 10^–4^	6.58 × 10^–5^	7.7 × 10^–3^
Both	17	32593665	A	0.72546	1.6 × 10^–4^	7.32 × 10^–5^	3.0 × 10^–2^
Both	21	45615741	G	0.394392	1.3 × 10^–4^	6.69 × 10^–5^	4.7 × 10^–2^
Both	7	50175654	G	0.573233	−1.7 × 10^–4^	6.62 × 10^–5^	1.2 × 10^–2^
Both	7	50304461	C	0.33364	−1.4 × 10^–4^	6.94 × 10^–5^	4.1 × 10^–2^
Crohn’s disease	12	6491125	G	0.646309	−1.6 × 10^–4^	6.93 × 10^–5^	1.8 × 10^–2^
IBD	7	2869985	T	0.697662	2.3 × 10^–4^	7.12 × 10^–5^	1.1 × 10^–3^
IBD	13	27531267	T	0.82362	2.2 × 10^–4^	8.58 × 10^–5^	1.1 × 10^–2^

Summary statistics of the known risk variants for Crohn’s disease and IBD in our microscopic colitis genome-wide association study for those that pass the nominal *p* value threshold of 0.05. Abbreviation: IBD, inflammatory bowel disease; GRS, genetic risk score.

## 4. Discussion

We have conducted a clinical and genetic case-control study of microscopic colitis in the UK Biobank. We confirm previously reported phenotypic associations with age, sex, coeliac disease, smoking status and PPIs. In a GWAS, we have confirmed recent reports of association with SNPs on the MHC 8.1 haplotype, indicating an immune component to the pathogenesis of microscopic colitis. Using genetic risk scores, we obtain results consistent with overlap in genetic risk factors for CD and IBD but not UC. This may suggest shared genetic pathways between these phenotypes.

This is the largest GWAS of microscopic colitis to date, and the first to look at genetic overlap between microscopic colitis and IBD by using a genetic risk score. The main limitation of the study is that the ICD10 coding in the UK Biobank only covers the first decimal place: K52.8. We acknowledge two limitations as a consequence: this code also includes eosinophilic gastritis and colitis, and we are unable to distinguish lymphocytic from collagenous colitis. However, eosinophilic gastritis and colitis are of very low prevalence [5.1 and 2.1/100 000 persons respectively]^18^ compared to microscopic colitis [103.0/100 000 persons].^1^

The UK Biobank ICD10 data rely on hospital coding data, which has limitations in terms of whether patients have had a coded diagnosis at hospital, and rely on the accuracy of hospital coding; however, their use is standard practice in literature performing UK Biobank GWASs. This study was performed using only white Europeans, and further studies are required to determine if the results are consistent across other ethnic groups. The UK Biobank also only includes patients between the ages of 40 and 69 years at recruitment, although this covers the peak age of onset for microscopic colitis. We are confident our main GWAS result is not a false positive due to the high MAF of the lead SNP and aligning closely with previous work, but we acknowledge that due to having under 500 cases, there may by many SNPs we were unable to detect due to low OR or MAF. A meta-analysis combining the results of this study and others may help to identify further associations.

To follow up on the strong association with PPIs, we performed a GWAS to find possible underlying pharmacogenetic associations, but there were no genome-wide significant SNPs. A follow-up study with a dedicated drug-exposed cohort would have greater power to detect such associations, as has been demonstrated in our recent study of thiopurine-induced myelosuppression.^19^

## Funding

S.E.J. is funded by the Medical Research Council [grant: MR/M005070/1]. M.N.W. is supported by the Wellcome Trust Institutional Strategic Support Award [WT097835MF]. A.R.W. is supported by the European Research Council grants: SZ-245 50371-GLUCOSEGENES-FP7-IDEAS-ERC and 323195. R.B. is funded by the Wellcome Trust and Royal Society grant: 104150/Z/14/Z. J.T. is funded by a Diabetes Research and Wellness Foundation Fellowship. The funders had no influence on study design, data collection and analysis, decision to publish, or preparation of the manuscript.

## Conflict of Interest

None declared.

## Author Contributions

H.G., J.G., N.K., T.A. and M.W. participated in the conception, design and coordination of the study. H.G., R.B. and M.W. performed data analysis. H.G. and M.W. participated in writing the paper. All authors assisted in the writing, reviewing and approval of the manuscript.

## Supplementary Material

jjz104_suppl_Supplementary_Material
