# Supplementary material for: Genome-Wide Association Study of Microscopic Colitis in the UK Biobank Confirms Immune-Related Pathogenesis
Source: J Crohns Colitis. 2019 May 24;13(12):1578–82. doi: 10.1093/ecco-jcc/jjz104 (PMC6903793; doi:10.1093/ecco-jcc/jjz104)
Supplement: jjz104_suppl_Supplementary_Material [file jjz104_suppl_supplementary_material.docx]

# $e^{\beta/ (\mu* (1 - \mu))}$

$$GRS=\sum_{i=1}^{N} \beta_{i}\cdot d_{i}$$

Supplementary Table 1

| **Class** | **Drug** | **Biobank Code** | **Cases** | **Controls** |
| --- | --- | --- | --- | --- |
| PPIs | Omeprazole | 1140865634 | 11.2% [54/483] | 6.4% [28,868/450,616] |
|  | Lansoprazole | 1140864752 | 7.7% [37/483] | 3.8% [17,065/450,616] |
|  | Esomeprazole | 1141177526 | 1% [5/483] | 0.35% [1,590/450,616] |
|  | Rabeprazole | 1141168584 | 0.4% [2/483] | 0.2% [842/450,616] |
|  | Pantoprazole | 1140929012 | 1% [5/483] | 0.2% [854/450,616] |
| SSRIs | Sertraline | 1140867878 | 0.6% [3/483] | 0.5% [2,121/450,616] |
|  | Paroxetine | 1140867888 | 0.8% [4/483] | 0.3% [1,539/450,616] |
|  | Fluvoxamine | 1140879544 | 0% [0/483] | 0.01% [29/450,616] |
|  | Fluoxetine | 1140879540 | 1.9% [9/483] | 1.2% [5,436/450,616] |
|  | Escitalopram | 1141180212 | 0.4% [2/483] | 0.2% [825/450,616] |
|  | Citalopram | 1140921600 | 2.1% [10/483] | 1.8% [8,187/450,616] |
| NSAIDs | Aceclofenac | 1140925806 | 0% [0/483] | 0.01% [54/450,616] |
|  | Aspirin | 1140868226 | 18.4 [89/483] | 13.7 [61,826/450,616] |
|  | Celecoxib | 1141176662 | 0.2% [1/483] | 0.1% [492/450,616] |
|  | Dexketoprofen | 1141164746 | 0% [0/483] | 0% [7/450,616] |
|  | Diclofenac Sodium | 1140878036 | 0% [0/483] | 0.07% [307/450,616] |
|  | Etodolac | 1140871188 | 0% [0/483] | 0.07% [335/450,616] |
|  | Etoricoxib | 1141180140 | 0% [0/483] | 0.09 [407/450,616] |
|  | Fenoprofen | 1140871226 | 0% [0/483] | 0% [3/450,616] |
|  | Ibuprofen | 1140871310 | 11% [53/483] | 13% [58,536/450,616] |
|  | Ketoprofen | 1140871506 | 0% [0/483] | 0.03% [135/450,616] |
|  | Mefenamic Acid | 1140871542 | 0% [0/483] | 0.1% [476/450,616] |
|  | Meloxicam | 1140926732 | 0.6% [3/483] | 0.3% [1,310/450,616] |
|  | Nabumetone | 1140875336 | 0% [0/483] | 0.05% [206/450,616] |
|  | Naproxen | 1140871462 | 0.6% [3/483] | 0.74% [3,353/450,616] |
|  | Piroxicam | 1140871666 | 0.2% [1/483] | 0.04% [158/450,616] |
|  | Sulindac | 1140871604 | 0% [0/483] | 0% [15/450,616] |
|  | Tenoxicam | 1140875346 | 0% [0/483] | 0% [13/450,616] |
|  | Tiaprofenic Acid | 1140871614 | 0% [0/483] | 0% [8/450,616] |
| Statins | atorvastatin | 1141146234 | 3.1% [15/483] | 3.4% [15,439/450,616] |
|  | fluvastatin | 1140888594 | 0.2% [1/483] | 0.05% [213/450,616] |
|  | pravastatin | 1140888648 | 0.4% [2/483] | 0.6% [2457/450,616] |
|  | rosuvastatin | 1141192410 | 0.8% [4/483] | 0.7% [29,44/450,616] |
|  | simvastatin | 1140861958 | 15.3% [74/483] | 11.9% [53,451/450,616] |

*Supplementary Table 1:* ***UK Biobank Medication Codes –*** *The treatment codes in UK Biobank used to define drug phenotypes.*

Supplementary Table 2


| CHR | POS | A1 | A1FREQ | BETA | SE | P |
| --- | --- | --- | --- | --- | --- | --- |
| 1 | 102300829 | A | 0.78544 | -0.00039 | 7.98E-05 | 1.20E-06 |
| 1 | 211852006 | C | 0.895625 | 0.000483 | 0.000107 | 6.80E-06 |
| 2 | 29002900 | A | 0.96978 | -0.00088 | 0.000192 | 4.50E-06 |
| 2 | 44211546 | A | 0.420328 | -0.00031 | 6.65E-05 | 3.90E-06 |
| 3 | 21580556 | T | 0.972868 | -0.0009 | 0.000201 | 7.60E-06 |
| 4 | 24338324 | C | 0.774897 | -0.00038 | 7.85E-05 | 1.40E-06 |
| 4 | 108478191 | T | 0.831665 | 0.000448 | 8.72E-05 | 2.80E-07 |
| 5 | 6222490 | C | 0.973156 | -0.00093 | 0.000203 | 4.60E-06 |
| 5 | 24256226 | C | 0.943636 | -0.00066 | 0.000143 | 3.60E-06 |
| *6* | *31355318* | *T* | *0.758228* | *-0.00042* | *7.64E-05* | *3.30E-08* |
| 6 | 65350792 | G | 0.83685 | 0.000415 | 8.86E-05 | 2.80E-06 |
| 6 | 104003704 | C | 0.929096 | -0.00057 | 0.000128 | 8.20E-06 |
| 6 | 106000790 | C | 0.951844 | -0.00082 | 0.000153 | 8.90E-08 |
| 7 | 25573204 | G | 0.789508 | -0.00039 | 8.04E-05 | 1.20E-06 |
| 7 | 47391321 | G | 0.970032 | -0.00092 | 0.000191 | 1.60E-06 |
| 8 | 12517525 | G | 0.969694 | -0.00097 | 0.000192 | 4.50E-07 |
| 11 | 11931183 | C | 0.950888 | -0.00071 | 0.000152 | 2.50E-06 |
| 11 | 105858586 | A | 0.911153 | -0.00055 | 0.000117 | 2.40E-06 |
| 13 | 54703907 | GCA | 0.974328 | -0.00107 | 0.00021 | 3.30E-07 |
| 14 | 41804188 | A | 0.520016 | -0.0003 | 6.55E-05 | 5.90E-06 |
| 15 | 64768700 | T | 0.972247 | -0.00102 | 0.000199 | 3.10E-07 |
| 16 | 26117039 | C | 0.871776 | -0.00048 | 9.80E-05 | 1.20E-06 |
| 17 | 6571439 | C | 0.964387 | -0.00088 | 0.000176 | 5.80E-07 |
| 20 | 2374455 | G | 0.869848 | -0.00046 | 9.80E-05 | 2.50E-06 |

*Supplementary Table 2:* ***GWAS Summary Statistics –*** *All risk loci with p<10^-5^ from the main GWAS, using all European Biobank Participants and BOLT-LMM. N=483 cases and 450,616 controls. SNPs have been condensed into genomic loci using FUMA* ^12^*. Full summary statistics can be found at the GWAS catalog at https://www.ebi.ac.uk/gwas/home.*

# Supplementary Table 3


| **Gene Set** | **N genes** | **Beta** | **Beta STD** | **SE** | **P** | **Pbon** |
| --- | --- | --- | --- | --- | --- | --- |
| GO bp:go digestive tract morphogenesis | 48 | 0.601 | 0.0303 | 0.133 | 3.02E-06 | 0.032 |
| GO bp:go morphogenesis of an epithelial fold | 15 | 0.802 | 0.0226 | 0.212 | 7.50E-05 | 0.799 |
| GO bp:go negative regulation of bmp signaling pathway | 41 | 0.493 | 0.023 | 0.135 | 0.000127 | 1 |
| GO bp:go regulation of calcium ion transmembrane transport | 116 | 0.279 | 0.0218 | 0.0769 | 0.000146 | 1 |
| GO bp:go prostate gland development | 42 | 0.471 | 0.0222 | 0.135 | 0.000243 | 1 |
| GO bp:go wnt signaling pathway calcium modulating pathway | 39 | 0.464 | 0.0211 | 0.133 | 0.000245 | 1 |
| GO mf:go r smad binding | 23 | 0.62 | 0.0216 | 0.179 | 0.000274 | 1 |
| GO bp:go regulation of calcium ion transmembrane transporter activity | 71 | 0.33 | 0.0202 | 0.0957 | 0.000283 | 1 |
| GO bp:go pattern specification process | 416 | 0.148 | 0.0217 | 0.0431 | 0.000304 | 1 |

*Supplementary Table 3:* ***MAGMA Gene-Set Analysis –*** *Top 10 results of a gene set enrichment analysis performed FUMA. We find a significant association for digestive tract morphogenesis when accounting for multiple testing.*

# Supplementary Figure 1


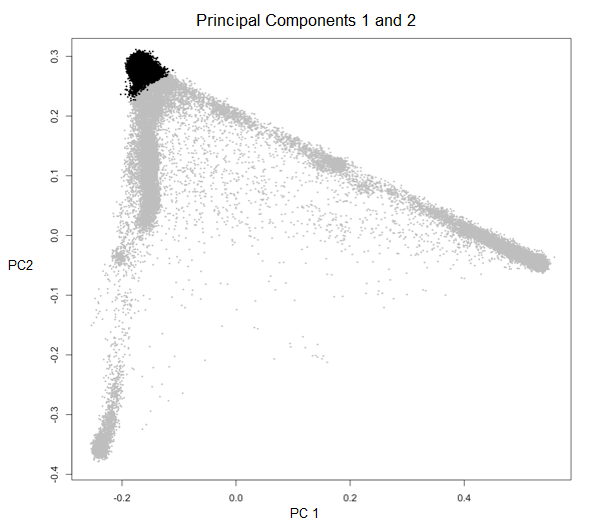

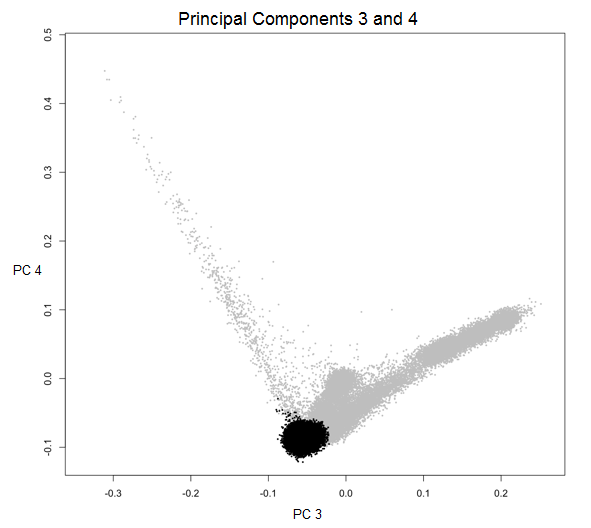


Derived European set of individuals after K-means clustering, anchoring on centres of PCs 1-4 for individuals self-reporting as from European population

# Supplementary Figure 2
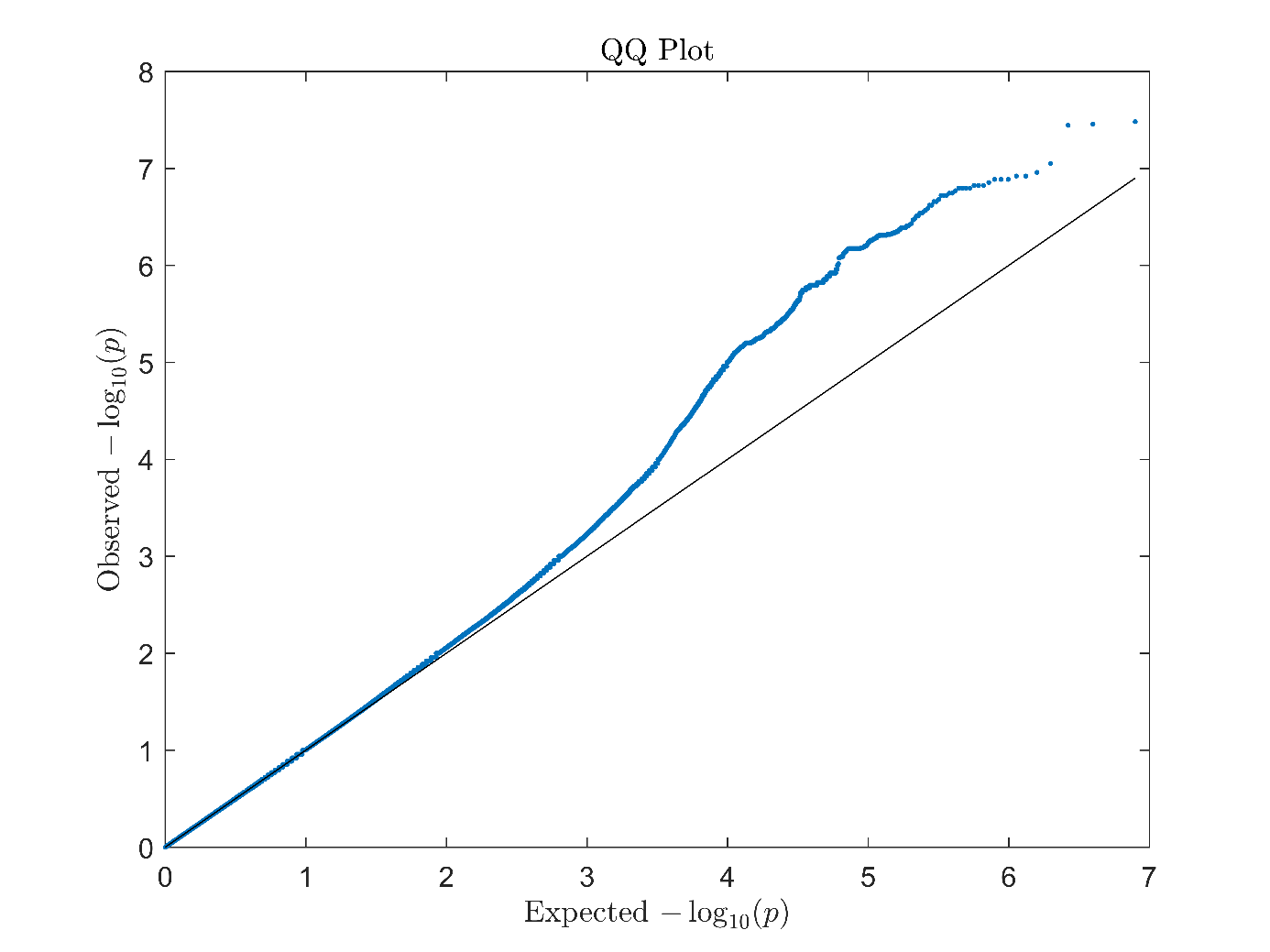


A QQ plot of our main GWAS result using BOLT-LMM on a Biobank Cohort with IBD and coeliac patients excluded. The median p value was 0.5000 and inflation factor λ=1.0000, showing no statistical inflation of p values.
